# Intravitreal Bevacizumab (Avastin) as an Adjuvant Treatment in Cases of Neovascular Glaucoma

**DOI:** 10.4103/0974-9233.53865

**Published:** 2009

**Authors:** Asaad A. Ghanem, Amr M. El- Kannishy, Ahmed S. El- Wehidy, Amira F. El-Agamy

**Affiliations:** From the Ophthalmology Center, Faculty of Medicine, Mansoura University, Egypt

**Keywords:** Avastin, Neovascular Glaucoma

## Abstract

**Purpose:**

To evaluate the effect of intravitreal bevacizumab (avastin) injection in cases of neovascular glaucoma.

**Study Design:**

Clinical case series.

**Materials and Methods:**

Sixteen eyes of 16 patients with rubeosis iridis and secondary glaucoma were administered intravitreal injection of bevacizumab (2.5 mg). The patients were followed for 2 months.

**Results:**

We noted partial or complete regression of iris neovascularization 1 week after injection of bevacizumab. Reproliferation of new vessels was detected in 25% of the cases after 2 months. The mean intraocular pressure (IOP) before injection was 28± 9.3 mm Hg under topical ß-blocker and systemic acetazolamide. One week after injection, the IOP decreased to 21.7± 11.5 mm Hg (5 cases without anti-glaucoma drugs, 6 cases with topical ß-blocker and 5 cases with both topical ß-blocker and systemic acetazolamide).

**Conclusion:**

Intravitreal bevacizumab (avastin) injection leads to regression of iris neovascularization with subsequent drop of IOP in eyes with neovascular glaucoma.

## INTRODUCTION

Neovascular glaucoma is a serious disorder, which occurs as a late complication of ischemic retinopathies. It is now well known that vascular endothelial growth factor (VEGF) plays a principal role in ocular conditions characterized by neovascularization.^1^ Activation of the VEGF-receptor pathway triggers signaling processes that promote growth of endothelial cells and their migration from preexisting vasculature.^2^ Bevacizumab (avastin) is a humanized monoclonal antibody that binds to all isoforms of VEGF.^3^ Regression of iris neovascularization after intravitreal injection of bevacizumab has been recently reported.^4^,^5^ This phenomenon encouraged many surgeons to use VEGF-inhibitors as a treatment for neovascular glaucoma. The aim of this study was to evaluate the effect of intravitreal bevacizumab injection in cases of neovascular glaucoma.

## MATERIALS AND METHODS

This is a prospective, observational clinical case series.

Sixteen eyes of 16 patients with rubeosis iridis and secondary glaucoma were included in this study. The primary cause for iris neovascularization was proliferative diabetic retinopathy (PDR).

Eight eyes had been previously treated with laser photocoagulation for their ischemic retina before inclusion in the study. Ocular examination included visual acuity assessment, slit-lamp biomicroscopy of the anterior segment, gonioscopy, applanation tonometry, indirect ophthalmoscopy and finally fluorescein angiography of iris neovascularization. The iris neovascularization was graded according to the iris angiography grading system.^6^ This grading included grade 0: the vessels that fill briefly with fluorescein are radial and do not leak; grade 1: the vessels appear more prominent and tortuous than normal, appear discontinuous, but do not leak fluorescein; grade 2: the vessels are more prominent, nonradial and leak fluorescein; grade 3: the vessels are more prominent, nonradial and leak early in the angiogram (by 20-30 seconds); and grade 4: individual vessels cannot be delineated in the early angiogram (by 20-30 seconds) and the iris appears as a diffuse opaque fluorescent sheet.^6^

All patients received intravitreal injection of bevacizumab (avastin), 2.5 mg in 0.1 mL. The following steps were used for injection: Eye speculum application, disinfection of conjunctival sac with povidone iodine 5% and then the intravitreal injection through lower outer quadrant 3.5-4 mm posterior to the limbus. Retinal photocoagulation was applied for cases with clear media as soon as possible after intravitreal injection. Cyclodestructive procedure cryotherapy application or glaucoma surgery (glaucoma drainage device implantation) was performed for eyes in which intraocular pressure (IOP) reduction was inadequate.

The patients were followed daily for 1 week and then weekly, for 2 months. Fluorescein angiography for iris neovascularization was done for every patient on the 2^nd^ postoperative day; and then at 1^st^, 4^th^, 6^th^ and 8^th^ weeks postoperatively.

The study was approved by the local ethics committee, and all patients signed informed consent before entering the study. All applicable institutional and governmental regulations concerning the ethical use of human volunteers were followed during this research.

## RESULTS

This study included 16 patients (10 males and 6 females); their ages ranged from 46 to 67 years (mean, 58.3± 6.2 years). The right eye was affected in 9 cases; and the left, in 7 cases. As shown in Table 1, iris neovascularization was graded 4 in 6 cases, 3 in 9 cases and 2 in 1 case. The preoperative IOP ranged from 17 to 48 mm Hg (mean, 28± 9.3 mm Hg) with systemic acetazolamide 750 mg/ three divided doses daily and topical β-blocker (betaxolol 0.5%, twice daily). The preoperative visual acuity ranged from LP (light perception) to 6/60.

**Table 1:** Clinical parameters before and after injection of bevacizumab (avastin)

Patient No.	Angiographic grading of iris new vessels			PAS		IOP in mmHg*				Best corrected visual acuity		Associated ocular conditions at inclusion
				
	Before injection	1 week	2 months	Before	After	Before injection	1 week	2 months	Other procedures	Before injection	At the end of follow up	
1	4	3	4		Stable	48	50	20^(T)^	Cyclocryo	LP	No LP	Significant** cataract
2	4	2	1		Stable	19	15^(T)^	15^(T)^		HM	HM	Significant**vit. hge.
3	4	1	0	No	No	28	18^(N)^	18^(N)^		1/60	1/60	
4	4	1	1	2/4	Stable	31	25	12^(N)^	A-G valve	C.F	2/60	
5	4	2	2	2/4	Stable	29	17	16		HM	CF	Significant**vit. hge.
6	4	0	0		Stable	21	18^(T)^	17^(T)^		C.F	1/60	
7	3	1	1	No	No	27	14^(N)^	15^(N)^		HM	2/60	Hyphaema
8	3	0	0	No	No	31	16^(N)^	14^(N)^		6/60	6/60	
9	3	1	1	4/4	Stable	45	45	12	Cyclocryo	LP	LP	
10	3	2	3	2/4		20	17^(T)^	22		CF	CF	
11	3	1	0	No	No	19	14^(N)^	15^(N)^		1/60	2/60	Mild vit. hge.
12	3	1	0	2/4	Stable	23	16^(T)^	18^(T)^		2/60	3/60	
13	3	1	1	No	No	17	15^(T)^	14^(T)^		1/60	1/60	
14	3	0	1		Stable	39	30	15^(N)^	A-G valve	CF	4/60	Mild vit. hge.
15	3	1	3		Stable	30	19^(T)^	28		CF	2/60	Mild vit. hge.
16	2	0	0			21	15^(N)^	16^(N)^		3/60	4/60

PAS = peripheral anterior synechiae, IOP = intraocular pressure, A-G valve = Ahmed glaucoma valve, vit. hge. = vitreous hemorrhage, LP = light perception, HM = hand motion, CF = counting fingers,

* IOP is described in this table under systemic acetazolamide (750 mg/day Cyclocryo: Cyclocryotherapy) and topical β-blocker but when the letter (T) is added, only topical, β-blocker is used and if the letter (N) is added, no antiglaucoma drugs are used.

** Significant cataract or vitreous hemorrhage is considered if the retina can't be visualized with indirect ophthalmoscopy through it.

**Figure 1:**
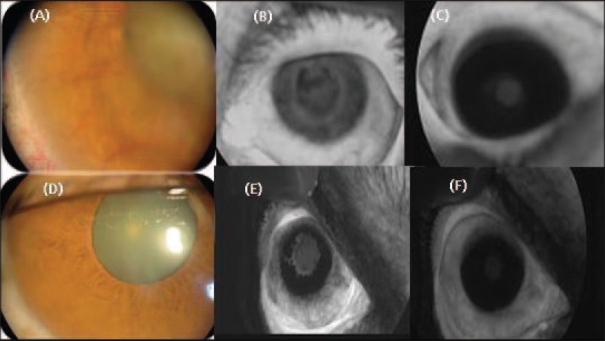
(A, B) Clinical photograph and early-phase iris angiography (grade 4), before injection of bevacizumab. (C) Late-phase angiography (grade 2) of the previous case, 1 week after injection. (D, E) Clinical photography and early-phase iris angiography (grade 3), before injection of bevacizumab. (F) Late-phase angiography (grade 0) of the previous case, 1 week after injection

After intravitreal injection of bevacizumab, the iris angiogram showed regression (partial or complete) of the new vessels with decreased vascular permeability in all cases (100%) within 1 week (Figure 1). After 2 months, regression was detected in 12 (75%) cases, which was complete (grade 0) in 6 (37.5%) eyes while 4 (25%) cases revealed regrowth of new vessels. This recurrence of iris neovascularization was detected 6 weeks after the injection.

Examination of the angle of the anterior chamber revealed complete regression of new vessels of the angle in all cases within 1 week. Peripheral anterior synechiae (PAS) remained unchanged during follow-up period except in 1 case (case 10), which showed more synechial angle closure together with reproliferation of new vessels in the angle and subsequent re-elevation of IOP.

One week after bevacizumab injection, the IOP decreased in 14 (87.5%) cases, while it remained persistently high in 2 (12.5%) cases (cases 1 and 9). These 2 patients underwent cyclodestructive procedure. (Cyclocryo procedure was done once in case no. 1 and twice in case no. 9.) The IOP decreased to below 20 mm Hg without the need of topical or systemic treatment in 5 (31.25%) eyes; while 6 (37.5%) eyes required topical β-blocker, and 1 (6.25%) eye was treated with topical β-blocker and systemic acetazolamide. In 2 (12.5%) cases (cases 4 and 14), though IOP decreased, it still remained above 20 mm Hg, and glaucoma valve implantation was done for each. Follow-up of the 12 cases with controlled IOP (< 20 mm Hg) for 2 months showed nearly stable IOP except in 2 cases (nos. 10 and 15), where the IOP re-elevated in the 7^th^ and 8^th^ weeks, respectively.

The mean IOP before injection was 28± 9.3 mm Hg under topical β-blocker and systemic acetazolamide. One week after injection, the mean IOP decreased to 21.7± 11.5 mm Hg (5 cases without anti-glaucoma medication, 6 cases with topical β-blocker and 5 cases with topical β-blocker and systemic acetazolamide). Two months after bevacizumab injection, the mean IOP was 16.7± 4.1 mm Hg. (Cyclocryo procedure was performed in 2 cases, and glaucoma valve implantation was done in 2 cases.)

At the end of follow-up, the visual acuity improved in 9 (56.25%) cases (56.25%), worsened in 1 (6.25%) case and remained at the same level in 6 (37.5%) cases. Improvement was mainly due to clearing of ocular media. Vitreous hemorrhage improved in 4 cases, and hyphema disappeared in 1 case. Only 1 case with significant vitreous hemorrhage (case no. 2) resisted clearance within the follow-up period. The effect of the drug on the macular area was not studied.

Clearance of ocular media together with improvement in pain and discomfort after intraocular injection allowed application of laser to the ischemic retina. Laser application was feasible in all except 3 cases (case 1, due to cataract; case 2, due to persistent vitreous hemorrhage; and case 9, due to persistent high IOP, which required 2 sessions of cyclocryotherapy procedure).

## DISCUSSION

In 2006, Kahook *et al.*^7^ studied the effect of intravitreal injection of bevacizumab (1 mg) in a case with neovascular glaucoma after failed intraocular pressure control with trans-scleral cyclophotocoagulation and panretinal photocoagulation. They reported regression of iris neovascularization with marked decrease of IOP (from 48 to 22 mm Hg). Another report^8^ recorded complete regression of iris and angle new vessels after intravitreal injection of bevacizumab (1.5 mg) in a patient with neovascular glaucoma. This regression remained for 8 weeks post-injection. The IOP in this patient decreased from 40 to 19 mm Hg with the same medications.

In this case series, the effect of intravitreal bevacizumab (2.5 mg) injection on 16 eyes with iris neovascularization and secondary glaucoma was studied. Angiography of iris neovascularization revealed regression (partial or more) in all cases (100%), which started from the 2^nd^ day post-treatment. This is consistent with the results of the studies by Oshima *et al.*^9^ and Iliev *et al.*^10^ In both studies, regression of iris neovascularization occurred in 100% of cases.

In our study, complete regression of iris neovascularization occurred in 37.5% of eyes within 2 months, while Oshima *et al.*^9^ recorded complete regression in 29% of eyes. On the contrary, Iliev *et al.*^10^ noticed complete regression of iris neovascularization at the end of follow-up period (range, 4 to 16 weeks) in 100% of eyes. It should be noted that in the last study,^10^ assessment and follow-up of iris neovascularization depended on clinical examination only and not fluorescein angiography.

In this study, reproliferation of iris neovascularization was detected in 4 (25%) cases at 6 weeks after the intravitreal injection. Oshima *et al.*^9^ reported recurrence of new vessels proliferation 2 months after a single intravitreal injection in 29% of eyes, which required a second injection of bevacizumab.

Iliev *et al.*^10^ reported a decrease of mean IOP from 36.0± 5.1 mm Hg before injection to 16.8± 3.4 mm Hg at the end of follow-up period (range, 4 to 16 weeks). In our study, the IOP decreased from a mean of 28± 9.3 mm Hg before injection to 21.7± 11.5 mm Hg 1 week after. All cases in our series showed a decrease of IOP except in 2 eyes (case. 1 and 9), in one of which the synechial angle closure was 8 clock hours; and in the other, nearly total. On the other hand, the 5 eyes (cases. 3, 7, 8, 11 and 16) which showed normalization of IOP without anti-glaucoma medications at one week post-injection had open angles in 4 cases and only one clock hour of PAS in one case. It is clear that the extent of synechial angle closure will determine the likelihood and the degree of IOP reduction after disappearance of iris neovascularization.

After intravitreal bevacizumab injection, the visual acuity improved in 9 (56.25%) cases, which was due to clearing of corneal edema, hyphema and/or vitreous hemorrhage. Rapid improvement of vitreous hemorrhage after intravitreal bevacizumab injection was observed in another study.^11^ Improvement of visual acuity also could be contributed to the effect of the drug on associated macular edema. Haritoglou *et al.*^12^ recorded a decrease of macular thickness and improvement of visual acuity in diabetic patients after intravitreal bevacizumab injection. However, the effect of the drug on the macular area was not studied in this work.

The current study demonstrates that intravitreal bevacizumab injection can successfully produce regression of iris neovascularization with subsequent decrease in IOP in cases of neovascular glaucoma.

Bevacizumab as a pharmacological treatment has the advantage of being rapid in effect but has the disadvantage of limited duration of action. Combining intravitreal bevacizumab injection with panretinal photocoagulation can offer both rapid and long-lasting effect.

Another advantage of bevacizumab is the ability to use this modality to treat eyes with opaque media which preclude treatment with laser photocoagulation.

Lastly, this study demonstrates that intravitreal bevacizumab injection may be the first choice, but not the only option in the treatment of neovascular glaucoma.
